# Imaging of Pheochromocytomas and Paragangliomas

**DOI:** 10.1210/endrev/bnae001

**Published:** 2024-01-11

**Authors:** Henri J L M Timmers, David Taïeb, Karel Pacak, Jacques W M Lenders

**Affiliations:** Department of Internal Medicine, Radboud University Medical Centre, 6525 GA Nijmegen, The Netherlands; Department of Nuclear Medicine, La Timone University Hospital, Aix-Marseille University, Marseille, France and European Center for Research in Medical Imaging, Aix-Marseille University, 13005 Marseille, France; Section on Medical Neuroendocrinology, Eunice Kennedy Shriver National Institute of Child Health and Human Development, National Institutes of Health, Bethesda, MD 20892-1583, USA; Department of Internal Medicine, Radboud University Medical Centre, 6525 GA Nijmegen, The Netherlands

**Keywords:** pheochromocytoma, paraganglioma, PET, SPECT, CT, MRI

## Abstract

Pheochromocytomas/paragangliomas are unique in their highly variable molecular landscape driven by genetic alterations, either germline or somatic. These mutations translate into different clusters with distinct tumor locations, biochemical/metabolomic features, tumor cell characteristics (eg, receptors, transporters), and disease course. Such tumor heterogeneity calls for different imaging strategies in order to provide proper diagnosis and follow-up. This also warrants selection of the most appropriate and locally available imaging modalities tailored to an individual patient based on consideration of many relevant factors including age, (anticipated) tumor location(s), size, and multifocality, underlying genotype, biochemical phenotype, chance of metastases, as well as the patient's personal preference and treatment goals. Anatomical imaging using computed tomography and magnetic resonance imaging and functional imaging using positron emission tomography and single photon emission computed tomography are currently a cornerstone in the evaluation of patients with pheochromocytomas/paragangliomas. In modern nuclear medicine practice, a multitude of radionuclides with relevance to diagnostic work-up and treatment planning (theranostics) is available, including radiolabeled metaiodobenzylguanidine, fluorodeoxyglucose, fluorodihydroxyphenylalanine, and somatostatin analogues. This review amalgamates up-to-date imaging guidelines, expert opinions, and recent discoveries. Based on the rich toolbox for anatomical and functional imaging that is currently available, we aim to define a customized approach in patients with (suspected) pheochromocytomas/paragangliomas from a practical clinical perspective. We provide imaging algorithms for different starting points for initial diagnostic work-up and course of the disease, including adrenal incidentaloma, established biochemical diagnosis, postsurgical follow-up, tumor screening in pathogenic variant carriers, staging and restaging of metastatic disease, theranostics, and response monitoring.

## Graphical Abstract

**Graphical abstract:**
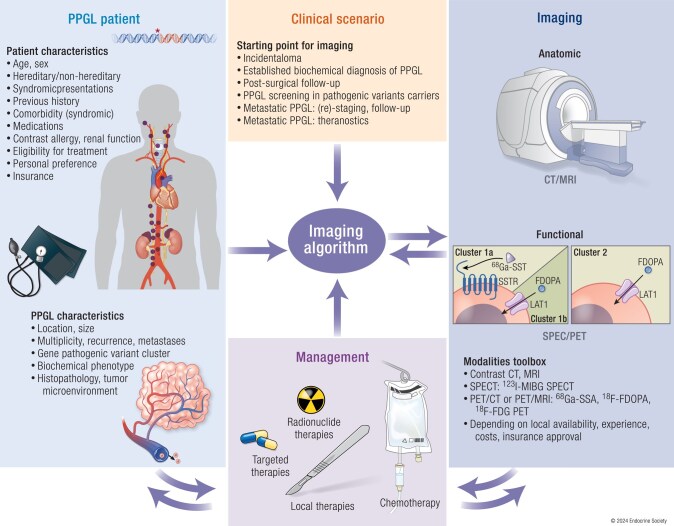

**Essential points**

- The genotypic and phenotypic heterogeneity of pheochromocytomas/paragangliomas (PPGLs) calls for different imaging strategies that serve the following goals: (1) locate, identify and delineate the tumor; (2) detect multifocality and metastases; (3) determine eligibility for local and systemic treatments; (4) monitor recurrence and evaluate response to treatment; (5) tumor surveillance in at risk patients due to germline pathogenic variants
- Anatomical imaging by contrast-enhanced computed tomography (CT) and magnetic resonance imaging (MRI), and functional imaging by positron emission tomography (PET)/CT and single photon emission computed tomography (SPECT)/CT are the essential modalities for the evaluation of patients with PPGLs. PET or SPECT radionuclides with the best diagnostic properties in this context are ^68^Ga-DOTA-somatostatin analogues (SSAs), ^18^F-FDOPA, ^123^I-MIBG, and ^18^F-FDG.
- Adrenal incidentaloma with low attenuation (<10 HU) on precontrast CT practically rules out PPGLs, whereas high postcontrast attenuation (>130 HU) points toward the diagnosis of pheochromocytoma (PCC). Contrast washout on the other hand is unreliable to distinguish between PCC and adrenocortical adenoma
- In patients with an established biochemical diagnosis of PPGL, anatomical imaging should be complemented by functional imaging in all patients besides when the risk of tumor multiplicity and metastases is low, namely, those without previous PPGL and hereditary syndrome, with an adrenergic biochemical phenotype and a single small adrenal PCC (<5 cm). Otherwise, the choice of ancillary imaging is mainly guided by biochemical phenotype and primary tumor location, such as ^68^Ga-DOTA-SSA PET/CT or ^18^F-FDOPA PET/CT for head and neck, ^18^F-FDOPA PET/CT or ^123^I-MIBG SPECT/CT for PCC, and ^68^Ga-DOTA-SSA PET/CT, ^18^F-FDOPA PET/CT, or ^18^F-FDG PET/CT for extra-adrenal PGL except (head and neck PGL).
- The principal role for imaging in the post-surgical follow-up is to identify residual or recurrent tumor in case of positive postoperative results of biochemical testing
- In carriers of succinate dehydrogenase pathogenic variants or other PPGL susceptibility genes, periodic tumor screening is indicated by means of biochemical testing in combination with imaging, in particular by whole body MRI and genotype-driven functional imaging
- ^68^Ga-DOTA-SSA PET/CT has become the cornerstone for staging and follow-up of metastatic PPGL, including its role besides ^123^I-MIBG SPECT in theranostics to evaluate eligibility for targeted radionuclide therapy

Pheochromocytoma (PCC) is defined as a catecholamine-producing tumor of the adrenal medulla. The extra-adrenal counterpart of this adrenal chromaffin cell tumor is referred to as paraganglioma (PGL), which develops from extra-adrenal paraganglia in specific locations in practically all body regions, often demanding the use of whole-body imaging to properly locate primaries as well as metastatic lesions. Furthermore, current imaging approaches to these tumors are guided by 2 additional important clinical points. First, PGLs are divided into 2 groups: head and neck paraganglioma (HNPGL) derived from parasympathetic paraganglia of the skull base and neck (eg, glomus: caroticum, jugulare, tympanicum, and vagale) as well as anterior/middle mediastinum and those that develop from sympathetic-associated chromaffin tissue in the abdomen (90%), less commonly from the pelvis, and rarely from the posterior mediastinum (2%). Second, PCCs and PGLs, here collectively referred to as PPGLs, although histologically similar, are highly heterogeneous with respect to their genetic landscape. In addition, they are associated with distinct clinical presentations, disease course and outcomes, which calls for different imaging algorithms to provide proper initial diagnosis as well as customized follow-up of patients with these tumors.

Based on differences in gene expression profiles, there are 2 main clinically relevant PPGL clusters guiding current imaging strategies for these tumors. Cluster 1 comprises genes related to the Krebs cycle/hypoxia signaling pathway: *SDHx* (succinate dehydrogenase subunits A-D and AF2), *VHL* (Von Hippel Lindau), *FH* (fumarate hydratase), *MDH2* (malate dehydrogenase), *GOT2* (glutamic-oxaloacetic transaminase), *SLC25A11* (solute carrier family member), and *EPAS1* (Endothelial PAS Domain Protein 1 also called *HIF2A* (hypoxia-inducible factor-2α) that are characterized by either high expression of cell membrane somatostatin receptor type 2 or L-amino acid transporter (LAT) and both are excellent imaging targets. Cluster 2 relates to genes involved in kinase signaling pathways: *RET* (responsible for MEN2 [Multiple endocrine neoplasia type 2]), *NF1* (Neurofibromatosis type 1), *TMEM127* (Transmembrane protein 127) and *MAX* (MYC-Associated factor X) and are characterized by high expression of cell membrane norepinephrine and/or LATs, also uniquely positioned in the imaging approach of these tumors. Furthermore, pathogenic variants in some of these genes may be responsible for well-defined syndromic features as is the case in VHL, MEN2, NF1, SDHx, and EPAS1-polycythemia syndromes, often requiring additional well-thought or modified imaging algorithms in order to detect tumors of specific developmental origin and clinical behavior (1). Lastly, apparently sporadic, multiple primary, metastatic, or recurrent PPGLs represent the category of tumors that require specific imaging approaches, some of them recently introduced.

As outlined above, the developmental, genotypic, and phenotypic heterogeneity and complexity of PPGLs pose endocrinologists and other health care professionals with an interesting but also challenging task in the work-up of a patient by selecting the most appropriate and regionally available imaging modalities that fulfill current clinical standards and are tailored to an individual patient. Thus, current and recommended optimal strategies for anatomical and functional (ie, molecular) imaging of patients with these tumors requires careful consideration of many relevant factors such as age, (anticipated) tumor location(s)/developmental origin, tumor size, previous history of PPGL, underlying hereditary, or other genetic conditions that may or may not be associated with syndromic presentation, biochemical phenotype, and specific clinical presentations including multifocality, recurrence, aggressiveness, and metastases. Furthermore, although not well proved at present, family history of PPGL in early age or metastatic disease, environmental factors (eg, chronic or intermittent hypoxia), developmental problems (eg, cardiac abnormalities), or therapies that result in immune suppression, constitute additional important aspects that may guide modification in imaging algorithms. Lastly, particularly in patients with PPGL, specific imaging signatures serve as a well-justified base for the use of systemic therapies, so-called theranostics as discussed later on, and very accurate tumor detection/responses on follow-up of patients with these tumors.

Thus, in this review based on the most up-to-date imaging guidelines, expert opinions, as well as the most recent and relevant studies and discoveries, we aim to define a personalized approach to imaging in patients with (suspected) PPGL from a practical clinical perspective.

## Imaging Toolbox

### Goals of Imaging

Computed tomography (CT), magnetic resonance imaging (MRI), and positron emission tomography (PET)/CT imaging are currently a cornerstone in the evaluation of patients with PPGL.

The general goals of imaging of any cancer, including PPGL, are to:

1. Locate a tumor.
2. Find imaging clues that assist in the differential diagnosis which is mainly based on anatomical location, marked enhancement on CT and MRI and positive uptake of PPGL-specific functional imaging tracers.
3. Detect multifocality or recurrence which often occurs in the setting of hereditary forms of PPGL.
4. Assess locoregional extension toward adjacent structures, including lymph nodes.
5. Assess distant metastases.
6. Assess feasibility of surgical removal or focused radiotherapies and chemotherapies.

To reach these goals, endocrinologists and other health care professionals should keep in mind that not all currently available imaging modalities are needed. There may be a sequential approach when choosing the modality to use in a specific patient (eg, to limit the amount of radiation, to get the most optimal results, to choose the most cost-effective approach as well as to recognize the availability of various imaging modalities in different hospitals, regions, and countries). Furthermore, it should be emphasized that imaging in a patient with suspected PPGL should only be initiated after a tumor is proven biochemically, except for the diagnosis of HNPGLs or other nonfunctional PPGLs, for instance in the setting of *SDHx* tumor screening, or when biochemical testing is considered unreliable.

### Anatomical Imaging

Nowadays, anatomical imaging has made huge progress in technical parameters, contrast dyes, as well as various options in how these agents can be administered to patients. When a clinician approaches a patient with a PPGL, CT, or MRI is considered as the first relatively easy and quickly available imaging modality in the evaluation of these tumors. Although both modalities precisely locate these tumors, they have very unique characteristics, providing different, although often complementary information about these tumors (Table 1). Each modality has also its limitations. Some expert-initiated guidelines have addressed their use for the initial diagnosis as well as for follow-up. Furthermore, it should be noted that previously used ultrasound in the evaluation of these tumors is not recommended anymore (due to its low sensitivity), except in the initial evaluation of young children and pregnant women with highly suspected PPGL. In some patients an incidental mass is discovered in the neck using ultrasound. A PGL can be suspected in the presence of the following criteria (2): hypoechogenicity, posterior acoustic enhancement, lack of fatty hilum, and particularly association with the carotid body or vagal nerve. Pulsed and color Doppler analysis can demonstrate high flow.

**Table 1.** Imaging modalities

	US	CT	MRI	SPECT/PET
Principle	Ultrasound wave (ie, echo)	X-ray attenuation	Magnetic properties of hydrogen nuclei (water)	Detection of Emission photons (gamma, annihilation)
Signal	Spontaneous contrast	Spontaneous contrast and following iodine	Spontaneous contrast and following gadolinium	Only following radiopharmaceutical injection
Acquisition protocols	Echo Doppler analysis	Noncontrast, contrast-enhanced (at 60 seconds), and delayed images (at 15 minutes)	T1, T2, and gadolinium-enhanced T1 Angiographic sequences for HNPGL Chemical shift imaging for adrenals	At approximately 1 hour postinejction for PET and 24-48 hours for SPECT
Advantages	Does not use radiation. Can quantify elasticity (elastography) and vascularity (doppler)	Very quick, less costly and higher spatial resolution than MRI, very accurate anatomical information. Excellent for evaluation of bone extension (eg, temporal bone) and metastatic disease including lungs	Higher soft tissue contrast than CT, Excellent for evaluation of HNPGL and metastatic disease (liver, soft tissues, bone marrow). Does not use radiation.	High sensitivity and high specificity (for specific molecular targets like NET LAT or SSTR)
Disadvantages	Limited anatomic field of exploration	Exposure to ionizing radiations	Higher motion artifacts than CT, costly, lower availability	Lower resolution than CT, variable performance across subtypes, costly, Exposure to ionizing radiations

Abbreviations: CT, computed tomography; HNPGL, head and neck paraganglioma; MRI, magnetic resonance imaging; US, ultrasound; SPECT, single photon emission computed tomography; PET, positron emission tomography.

#### Computed tomography

CT is the first anatomic imaging modality used in most patients with these tumors due to its broad availability, very short scanning time (whole body imaging can be done in a few minutes), and several advantages over MRI such as higher spatial resolution and lower motion artifacts (Table 1). There are several scanning protocols that are used for the detection of PPGL. When a PPGL is strongly suspected (by positive biochemical testing), contrast administration is mandatory (3). The acquisition protocol for an adrenal mass usually consists of 3 series—noncontrast, contrast enhanced (at 60-75 seconds after contrast is given, ie, portal venous phase, or at 120 seconds, ie, nephrogenic phase), and delayed images (at 15 minutes after contrast is given) of the abdomen. Contrast agent is given intravenously, while oral contrast administration is usually not necessary. Slice thickness of 3 mm or less is highly desirable to avoid volume averaging. The amount of administered iodine contrast should be adjusted according to the patient's body weight. Measurements of precontrast and 15 minutes postcontrast densities (expressed in Hounsfield units, HU), as well as analysis of delineation and mass composition are important for characterization.

**Figure 1.**
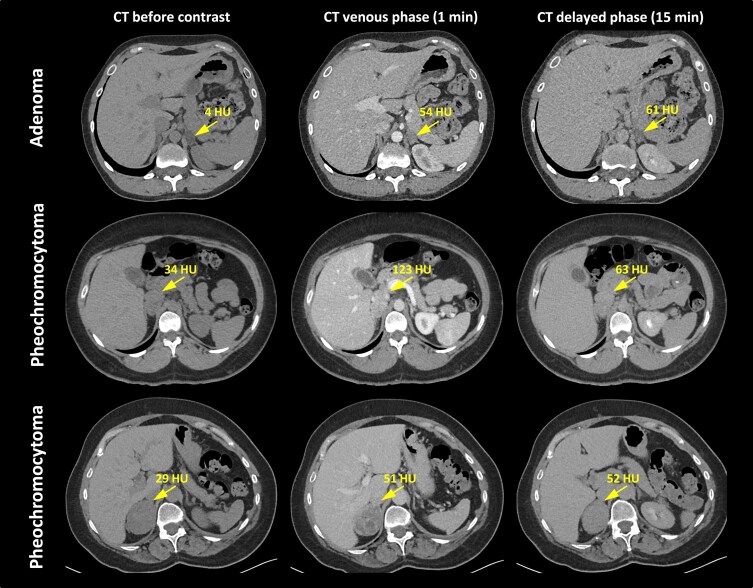
CT of PCC vs adenoma. CT images of a patient with left adrenal adenoma with low precontrast attenuation and lack of contrast washout (upper panels); a patient with right PCC with high postcontrast attenuation (123 HU) and high absolute (67%) and relative (49%) contrast washout (middle panels); a patient with right PCC displaying lack of contrast washout (lower panels). The adrenal lesions are marked by arrows. Abbreviations: CT, computed tomography; HU, Hounsfield units.

PPGL is usually a well-delimited solid mass with an attenuation of greater than 20 HU before contrast and a marked enhancement after contrast administration due to their abundant vascular supply (Fig. 1). Any adrenal lesion that enhances stronger than 130 HU on CT after contrast is most likely a PCC. In addition, enhancement can be heterogeneous or there may be no enhancement due to cystic or degenerated regions within the lesion (4, 5). Contrast-enhanced images can be particularly useful in the setting of incidentaloma or in rare circumstance where a PCC contains fat with low attenuation values (6-11). PCCs may demonstrate varying washout patterns and can be mistaken for lipid-poor adenoma as discussed in detail below. Of note, the presence of fatty areas (−10 to −100 HU) is uncommon in PCC.

Extra-adrenal retroperitoneal PGLs are typically para-aortic soft tissue masses with either homogenous enhancement (usually >20 HU) or central areas of low enhancement, eg, due to necrosis. The organ of Zuckerkandl PGL can be typically found near the origin of the inferior mesenteric artery or near the proximal common iliac arteries.

**Figure 2.**
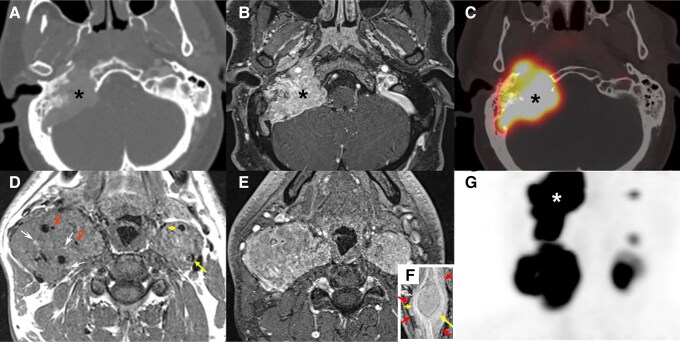
CT, MRI, and ^68^Ga-DOTATOC PET/CT of multifocal *SDHD*-related HNPGLs. Temporal bone CT (A), MRI (B, D, E), ^68^Ga-DOTATOC PET/CT (C, F). Right jugular PGL (A-C; asterisk, stage Fisch Di) with bilateral shamblin III carotid PGLs (D, E). Note the salt and pepper appearance on T1 (D, salt/white arrows = hemorrhage foci; pepper/red arrows = flow voids) and lyre sign (F, long yellow arrow: internal carotid artery, short yellow arrow: external carotid artery, short red arrows showing the limits of the PGL). On ^68^Ga-DOTATOC PET/CT: multiple SSTR-expressing PGL. Note the 2 left small foci corresponding to a small vagus nerve and a jugular PGL, respectively.

For HNPGLs, CT following contrast administration demonstrates a well-delimited mass with marked enhancement due to dense vascular supply. Therefore, angiography by CT is very sensitive for differentiating PGLs from other mimicking lesions. Carotid and jugular vessel displacement can be useful for distinguishing carotid body PGLs from vagal nerve PGLs. Regarding carotid body PGLs, anatomical imaging shows a “lyre sign” (Fig. 2), which refers to the splaying of the internal carotid artery and external carotid artery by the tumor. These findings differ from vagus nerve tumors that provoke internal carotid artery and internal jugular vein splaying without displacement of the external carotid artery. CT also provides indispensable information for evaluating temporal bone extension.

#### Magnetic resonance imaging

Conventional MRI is now becoming the anatomic imaging of the first choice for the evaluation of patients with HNPGL as well as for patients with a very high likelihood of developing these tumors (eg, carriers of PPGL susceptibility genes) (12). The current justification of this choice is that MRI with gadolinium contrast does not expose a subject to any radiation, it provides excellent contrast to noise ratio within soft tissue, and it allows to perform MR angiography sequences. Similar to CT scanning it can be performed as a whole-body imaging modality. However, there are some drawbacks related to the use of this imaging modality such as (1) in contrast to whole-body CT scan, whole-body MRI most often cannot be done in 1 setting and patients must come to an imaging facility at least twice, sometimes 3 times; (2) although there is no exposure to radiation, the use of gadolinium may very rarely cause allergic reactions and side effects (eg, pulmonary fibrosis), while gadolinium may accumulates within the brain (13). The consequences of this accumulation, especially in children, are currently unknown; (3) MRI can be suboptimal in patients with a previous operation in the presence of surgical clips; (4) MRI has lower spatial resolution when than CT, it is suboptimal for the evaluation of lung masses, and usually suffers from more imaging artifacts than CT; and (5) unlike CT ferromagnetic materials may preclude evaluation by MRI evaluation in many patients (Table 1). Noncontrast MRI, although recommended by some physicians, is usually suboptimal and often a clinically impractical method of evaluation in patients with PPGL with a suspected mass(es), although it can be used in surveillance in carriers with PPGL pathogenic variants (12).

**Figure 3.**
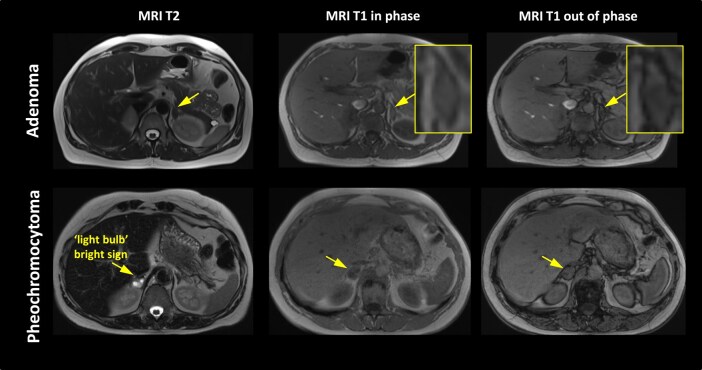
MRI of PCC vs adenoma. MRI images of a patient with left adrenal adenoma with loss of signal (“chemical shift”) in T1 out of phase (upper panels); MRI images of a patient with right PCC with “light bulb” bright sign an no loss of signal in T1 out of phase (lower panels). The adrenal lesions are marked by arrows. Abbreviations: MRI, magnetic resonance imaging.

**Figure 4.**
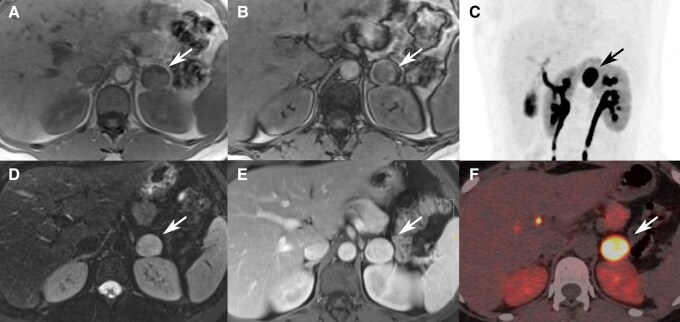
MRI and ^18^F-DOPA PET/CT of PCC. MRI (A, B, D, E), ^18^F-DOPA PET/CT (C, F). Homogeneous left adrenal mass hypointense on T1 MRI without loss of intensity between the in-phase (A) and out of phase images (B), intermediate high signal on T2 images (D), with hypervascular pattern at arterial phase (E). High tumor DOPA uptake on PET imaging (C: MIP image, F: axial PET/CT fused image).

There are several protocols regarding how MRI is used in the evaluation of these tumors. For an abdominal MRI, anatomic coverage should extend from the diaphragm to the aortic bifurcation. A pelvic or thoracic MRI can be added to detect extra-adrenal PGLs, if complete coverage is desired. For HNPGLs, MRI acquisition should cover the entire head and neck region. First, assuming that contrast MRI is used, T2-weighted and precontrast and postcontrast T1-weighted imaging are usually performed because they allow precise anatomical localization of the lesions. T1-weighted MRI enhances the signal of the fatty tissue and suppresses the signal of the water. T2-weighted MRI enhances the signal of the water. Gadolinium enhancement has predominant effects on T1 and enhances MR sensitivity. PPGLs usually demonstrate a low signal on T1-weighted images with flow voids leading to a “salt and pepper” appearance, usually described for HNPGL (Fig. 2). The “pepper” component represents the multiple areas of signal void, interspersed with the “salt” component, which is seen as hyperintense foci (due to slow blood flow). The classical “light-bulb” bright sign on T2-weighted MRI of PPGL (Fig. 3) is observed in about two-thirds of cases (14-18). Furthermore, other masses or pathologies other than PPGL can also be present with a “light-bulb” feature including cysts, lipid-poor adenoma and metastatic lesions. PPGL typically demonstrates avid contrast enhancement following gadolinium injection regardless of their location, similar to enhancement on CT. However, PPGLs may show variable enhancement depending on the presence of heterogenous elements, necrosis, cysts, and/or hemorrhage. Furthermore, as previously stated and in contrast to adenomas, PPGL do not usually contain fat and thus maintain their signal on opposed-phase gradient-echo images (chemical shift) (Figs. 3 and 4) (14).

Nevertheless, for PCC and sympathetic PGL, contrast-enhanced MRI serves as a valuable imaging modality and it is now preferred over CT to prevent or reduce radiation exposure, for instance in children and pregnant women, as well as in patients who require frequent follow-up scanning (eg, those with indolent metastatic disease or slow-growing inoperable tumors). MRI is also the anatomic imaging of choice for liver assessment as well as cardiac PGLs but not for the assessment of lung primary/recurrent PGLs or metastases.

MRI also has an important role in the evaluation of HNPGLs. Arterial tumor feeders can be seen as hyperintense vessels on MRI with angiography sequences. The latter can be performed without (“time of flight”) or with gadolinium injection in 3D or 4D mode. These sequences have very high sensitivities and specificities for HNPGL (19-22). There is a recent trend of using 4D time resolved MR angiography. Fusing images between MR angiography and T1-weighted images (especially with fat-saturated sequence) are particularly informative for localizing these tumors and describing their relationships with adjacent structures. MRI offers unique information for tumor delineation and is the preferred modality for assessing tumor extension according to the recommended classifications (ie, Fisch and Mattox's or Glasscock and Jackson's for tympanic and jugular PGL, Netterville's for vagal PGL, and Shamblin's for carotid body PGL). Estimation of tumor dimensions is usually done by measurement of 2 or 3 perpendicular diameters. This can be however suboptimal in the evaluation and surveillance of some HNPGL that often exhibit complex geometrical shapes with potential vascular extensions such as intraluminal extension in the venous sinus and jugular bulb.

Diffusion-weighted imaging is dependent on tissue cellularity and may be useful in tumor characterization, but would require further evaluation in PPGL (23). MR spectroscopy is an emerging technique that has shown promise in the assessment of succinate content in PPGL and other tumors that may coexist in the setting of SDHx (24-28), as discussed later on in this paper.

### Functional Imaging

Functional imaging represented by single photon emission computed tomography (SPECT) and positron emission tomography (PET) are nuclear medicine imaging techniques based on the use of radioisotope-based molecular imaging probes (radiopharmaceuticals or radiotracers). Unlike CT and MRI, nuclear imaging provides functional (ie, molecular information). Functional imaging of PPGLs has a pivotal role in precision medicine in their localization as well as specific characterization since these tumors possess some unique PPGL cell membrane characteristics. Therefore, functional imaging of these tumors become paramount in their assessment, not only before any operation but also in their follow-up as well as for therapeutic plans. Furthermore, functional imaging has an advantage compared to anatomic imaging since it is done as 1-time whole-body scan, covering even brain as well as part of lower extremities. It is a relatively short 1-time procedure compared to MRI and it is done in scanners that are open to avoid problems in claustrophobic/anxious patients.

#### SPECT vs PET

SPECT is cross-sectional (transverse) nuclear imaging using single-photon emitting radiopharmaceuticals such as ^123^I, ^131^I, ^111^In, and ^177^Lu. Hybrid cameras (eg, combining SPECT with CT) increase image quality and improve reader confidence. PET imaging is based on detecting 2-time–coincident high-energy photons from the emission of a positron-emitting radioisotope (eg, ^18^F, ^68^Ga, ^64^Cu). When compared with SPECT, PET technology provides better image resolution, less attenuation (due to higher photon energy) and scatter artifacts, and, consequently, superior quantitative and diagnostic capabilities (Table 2). Additionally, PET has higher sensitivity and has an extensive list of tracers, making PET a versatile and powerful tool for clinical and research applications. There are some drawbacks of PET vs SPECT, such as higher financial costs and short half-lives of PET isotopes that require a dedicated environment. The use of SPECT is currently limited to metaiodobenzylguanidine (MIBG) scan for selecting patients who are likely to benefit from MIBG therapy or ^111^In-labeled somatostatin receptor (SSTR) scintigraphy if PET tracers are not available. In the recent years, PET has become a key multimodality molecular imaging technique in the assessment of PPGL.

**Table 2.** Radionuclide imaging of PPGL

Ligands	Targets	Acquisition protocols	Specificity for PPGL	Indications	Drawbacks
^123^I-MIBG SPECT/CT	NET	Thyroid blockade Images acquired after 24 hours	High	Sporadic PCC Determine eligibility for ^131^I-MIBG therapy in mPPGL	Lower resolution and sensitivity compared to PET, uptake by healthy adrenal glands
^18^F-FDG PET/CT	Glucose transporters (mostly type 1) and hexokinase	Fast for 6 hours Images acquired at 1 hour	Low	*SDHx* PPGL (if SSTR not available)	High BAT uptake in norepinephrine secreting PPGL, lack of specificities
^18^F-FDOPA PET/CT	LAT1/2 and AADC	Fast for 3 hours Images acquired at 1 hour	High	PCC (sporadic, *VHL*, *RET*, *NF1*, *MAX*, *FH*, *EPAS1*) and *SDHD/C* HNPGL	Costly, limited availability
^68^Ga/^64^Cu-DOTA-SSA PET/CT	SSTR (mostly type 2)	Images acquired at 1 hour	Moderate	Metastatic PPGL and HNPGL (sporadic and *SDHx*) Determine eligibility for SSTR-targeted radionuclide therapy in mPPGL	Costly, uptake by healthy adrenal glands

Abbreviations: AADC, aromatic amino acid decarboxylase; BAT, brown adipose tissue; CT, computed tomography; EPAS1, endothelial PAS domain-containing protein 1; FDG, fluorodeoxyglucose; FDOPA, fluorodihydroxyphenylalanine; HNPGL, head and neck paraganglioma; LAT1/2, L-amino acid transporter 1/2; MIBG, metaiodobenzylguanidine; NET, norepinephrine transporter; PET, positron emission tomography; PPGL, pheochromocytoma/paraganglioma; SDHx, succinate dehydrogenase pathogenic variants; SPECT, single photon emission computed tomography; SSA, somatostatin analogue; SSTR, somatostatin receptor; VHL, von Hippel–Lindau.

In contrast to MRI or CT, PET scanners have intrinsic limitations, meaning that lesions smaller than 3 to 5 mm are hard to detect. This, however, depends on signal to noise ratio that can be increased with new PET devices. Furthermore, the very accurate shape and size of a lesion depicted on PET cannot even be well assessed if PET is combined with a nondiagnostic CT, this latter done without iodine contrast injection. Therefore, for any surgical or other therapeutic approaches (eg, radiofrequency ablation, etc.), PET imaging must be done together with either regular diagnostic contrast-enhanced CT or MRI. There is also a drawback in the availability of some PPGL-specific radiopharmaceuticals used for PET/CT in the evaluation of these tumors. On the other hand, during the last 5 to 10 years, functional imaging of PPGL is witnessing huge progress and improved worldwide availability of some radiopharmaceuticals, especially SSTR-targeted PET. Finally, the methodological protocol for PET/CT imaging is usually straightforward not demanding various options as seen in CT or MRI (eg, contrast vs no contrast, specific sequences). Cost of these scans is comparable with the cost of the whole-body CT or MRI. The most important limitation of PET scanning are the chemical half-life of various radioisotopes and their production that can be done either on site (eg, using generators for ^68^Ga or cyclotrons for ^18^F (Table 2). Due to the advantages of ^18^F over ^68^Ga (can be produced by cyclotrons in large amounts, longer half-life, better spatial resolution due to better physical properties), several ^18^F-labeled tracers have been developed for SSTR PET/CT of neuroendocrine tumors and have shown excellent results. This will help overcome a number of important hurdles and make these imaging modalities more widely available.

#### ^123^I-metaiodobenzylguanidine

**Figure 5.**
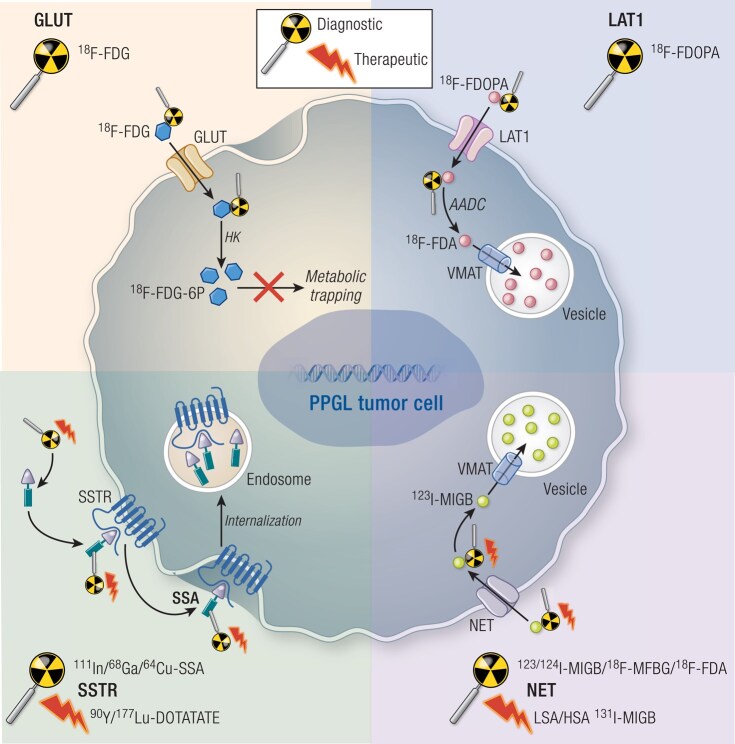
Radionuclide targets in PPGL. Functional imaging and theranostics of PPGL relies on radionuclide targeting of transporter systems and receptors on the tumor cell membrane. These targets include the glucose transporter (left upper panel), L-amino acid transporter 1 (right upper panel), the NET (right lower panel), and the somatostatin receptor (left lower panel). After entry into the cell through transporters, radionuclides are either metabolized and trapped (^18^F-FDG, left upper panel) or stored in catecholamine vesicles either directly (eg, ^123^I-MIBG, right lower panel) or after metabolization (^18^F-FDOPA, right upper panel). Alternatively, somatostatin analogue radionuclides bind to their receptor(s) and are subsequently internalized (left lower panel). Abbreviations: AADC, aromatic amino acid decarboxylase; FDA, fluorodopamine; FDG(-6P), fluorodeoxyglucose (-6 phosphate); FDOPA, fluorodihydroxyphenylalanine; GLUTs, glucose transporters; HK, hexokinase; LAT1, L-amino acid transporter 1; MFBG, meta-fluorobenzylguanidine; MIBG, metaiodobenzylguanidine; NET, norepinephrine transporter; PPGL, pheochromocytoma/paraganglioma; SSA, somatostatin analogue; SSTR, somatostatin receptor; VMAT, vesicular monoamine transporter.

^123^I-MIBG is an iodinated analogue of guanidine, which is structurally similar to norepinephrine. It is taken up by cells via the norepinephrine transporter (NET) on the PPGL cell membrane and stored within the neurosecretory granules via vesicular monoamine transporters 1 and 2 (Fig. 5). Both uptake processes are expressed in PPGL tissue, resulting in the detection of these tumors. Nevertheless, since the uptake of MIBG depends on the presence of 2 transporter systems, it is expected that there are many drugs that can alter or even inhibit this uptake resulting in no detection of PPGL (Table 2). Thus, proper preparation of a patient, usually by temporary discontinuation of drugs interfering with MIBG uptake and retention is usually recommended but in clinical practice difficult to accomplish (eg, opioids, tricyclic antidepressants, sympathomimetics, antipsychotics, and certain antihypertensive agents such as labetalol (29-36)). On the other hand, the calcium channel blocker nifedipine can cause prolonged MIBG retention in PPGLs (36). It is also known that in patients with high catecholamine levels (especially norepinephrine) there can be some competition between endogenous norepinephrine and administered MIBG on the cell membrane NET system. Since MIBG is given with radioactive iodine, all patients receiving this radiopharmaceutical must undergo iodine thyroid blocking by the oral administration of potassium iodide.

Nowadays, imaging with ^123^I-MIBG has largely replaced imaging with ^131^I-MIBG because of its lower radiation dose and superior imaging quality. ^123^I-MIBG scans are usually obtained 24 hours after tracer injection although delayed (eg, 48 hours) images can be occasionally obtained to, for instance, distinguish between bowel activity and PGL. When reading ^123^I-MIBG scans, it should be noted that physiological uptake of ^123^I-MIBG includes myocardium, salivary glands, lacrimal glands, thyroid, liver, lungs, adrenal glands, bowel, and uterus (during menstruation).

^123^I-MIBG scintigraphy has a sensitivity of about 83% to 100% and a very high specificity of about 98% to 100% in detecting PCC. However, its sensitivity has been revised downwards in comparative studies with PET imaging, especially for small tumors, *SDHx* PPGL (particularly *SDHB*), metastatic PPGLs, and HNPGLs (37-40). In more aggressive (eg, fast growing tumors) or metastatic lesions, this is most likely due to lower expression of tumor cell membrane transporter systems. Therefore, now in clinical practice, ^123^I-MIBG scintigraphy is not recommended as a functional imaging study of first choice as it used to be previously and it is usually recommended only in patients in whom inoperable or metastatic PPGLs are found and radiotherapy with ^131^I-MIBG is strongly considered. Thus, before deciding to use ^123^I-MIBG scintigraphy, CT, or MRI results should be available, often together with genetic testing results. The presence of *SDHx* pathogenic variants usually precludes the use of this imaging modality from the initial imaging algorithm of these tumors (39), unless it is the only option locally available.

#### ^18^F-fluorodeoxyglucose

First, it should be noted that ^18^F-fluorodeoxyglucose (FDG) is not specific of PPGL since positive images simply reflect glucose uptake and its metabolism by cells, including any cancer cells that have a high demand for glucose. ^18^F-FDG is taken up by tumor cells via glucose membrane transporters and is phosphorylated by hexokinase into ^18^F-FDG-6P. ^18^F-FDG-6P does not follow further enzymatic pathways, thus it is trapped in a cell and its subsequent accumulation serves as basis for positive images. Before ^18^F-FDG PET/CT is performed, patients must fast for at least 6 hours. Patient with diabetes, which is not uncommon in PPGL, require specific instructions for tight glucose control. Scans are usually obtained at 60 minutes (45-90 minutes) after injection (Table 2).

Although ^18^F-FDG PET/CT lost its priority in the initial imaging of PPGLs, particularly in the assessment of metastatic disease, its role in the imaging algorithm of these tumors is still important. ^18^F-FDG PET positivity is present in about 80% of PPGL with usually low to moderate uptake. However, it is remarkable that some PPGLs, associated with a TCA defect (here those particularly associated with *SDHx* pathogenic variants), exhibit the highest ^18^F-FDG uptake values among other hereditary or nonhereditary PPGLs (39, 41-43). Nevertheless, previous data suggest that ^18^F-FDG PET/CT is inferior to SSTR targeted PET/CT in the evaluation of metastatic *SDHx* and nonhereditary PPGLs, HNPGLs, and of sporadic, recurrent, or multiple PPGLs. It is also inferior for the detection of other PPGLs, such as those related to the presence of *FH* and *HIF2A* pathogenic variants in which ^18^F-fluoro-dihydroxyphenyl-alanine (^18^F-FDOPA) PET/CT is the imaging modality of a choice. Nevertheless, ^18^F-FDG PET/CT plays an important role in the evaluation of PPGLs in situations where these tumors becoming very aggressive, such as rapidly growing with the occurrence of new metastatic lesions or rapid growth of already existing metastases. In such situations, ^18^F-FDG PET/CT is often superior to SSTR targeted PET/CT and such rapid “shift” in the imaging pattern reflects tumor aggressivity and dedifferentiation and requires urgent treatment, usually using chemotherapeutic approaches. Finally, it should be noted, that there are several potential differential diagnoses that should be considered in cancer patients in cases of ^18^F-FDG-avid adrenal or extra-adrenal masses such as lymphoma, adrenocortical carcinoma, adrenal oncocytoma, and metastasis.

#### ^18^F-fluorodihydroxyphenylalanine

Dihydroxyphenylalanine is a precursor of catecholamines. ^18^F-FDOPA is taken up through neutral LATs and is also expressed on cell membranes of PPGLs. Upon the entry into a tumor cell, ^18^F-FDOPA is decarboxylated into ^18^F-fluorodopamine by aromatic L-amino acid decarboxylase, and then stored into intracellular vesicles. There is no reported drug interaction in PPGL that could interfere with ^18^F-FDOPA uptake. It is advised that patients should fast for at least 3 to 4 hours prior to injection, since certain amino acids can competitively inhibit ^18^F-DOPA influx into tumor cells. Scans are usually obtained 30 to 60 minutes after tracer injection (Table 2). PPGLs classically exhibit a marked ^18^F-DOPA uptake, especially those associated with polycythemia, those that belong to cluster 2 PPGLs, and HNPGLs (Fig. 4). ^18^F-DOPA PET/CT has a sensitivity approaching 100% and a very high specificity (>95%) for these tumors; however, it is substantially lower for metastatic tumors (37, 39, 44-51). The main advantage over other tracers (eg, in comparison to ^123^I-MIBG and labeled-SSA) relies on the low to moderate uptake by healthy adrenals, thus allowing more specific detection of PCC than ^123^I-MIBG. The main drawback of this radiopharmaceutical is that it is not approved or routinely available in most countries.

#### Somatostatin analogs

In recent years, dodecane tetraacetic acid (DOTA) conjugated somatostatin analogues (SSAs) such as ^68^Ga-DOTATATE and ^64^Cu-DOTATATE are applied as PET radiopharmaceuticals and have become paramount in the diagnostic localization of PPGL since these tumors express SSTRs, particularly type 2. Thus, currently this type of functional imaging is in world-wide use, especially using ^68^Ga-DOTATATE PET/CT. Before the scan is performed, patients do not need to fast. Scans are usually obtained at 60 minutes (45-90 minutes) after tracer injection (Table 2). SSA PET/CT was found to be more sensitive than other tracers in any *SDHx* PPGLs and sporadic or hereditary HNPGLs and metastatic PPGLs (lesion-based detection rate approaching 100%) (52-58). It was suggested to be less sensitive for abdominal PPGL (59), although comparative studies are still lacking. SSA PET/CT can be falsely positive in metastatic lymph nodes due to various other cancers, meningiomas, some central nervous system conditions, inflammatory processes, and rare conditions such as fibrous dysplasia (60). ^68^Ga-DOTATATE PET/CT has inherent limitations. Particularly, a short half-life of 68 minutes requires that it be locally produced via a generator and used on-site, limiting availability of this radiopharmaceutical to large medical centers. Recently, ^64^Cu-DOTATATE has been approved by the Food and Drug Administration (FDA) and has similar clinical value than ^68^Ga-DOTA-SSA. Despite some potential physical advantages of ^64^Cu compared with ^68^Ga such as longer half-life (12.7 hours vs 68 minutes) that allows to perform delayed images and dosimetry studies, both ^68^Ga-DOTATATE and ^64^Cu-DOTATATE could be considered as clinically interchangeable for SSTR imaging.

## Clinical Scenario I: The Role of Imaging at the Initial Diagnosis of PPGL

### Biochemical Diagnosis

Biochemical screening for PPGL is indicated in (1) patients with signs and symptoms or cardiovascular events, highly suggestive of acute spells (sometimes called attacks or storms) of catecholamine excess; (2) during follow-up of previous PPGL; (3) patients with the presence of germline pathogenic variants in one of the PPGL susceptibility genes or in those with a family history of PPGL; (4) Patients with syndromic presentations associated with PPGLs; (5) patients with an adrenal incidentaloma (61). For many years, most PPGLs were diagnosed based on clinical suspicion, while only less than 10% patients were diagnosed based on the findings of an adrenal incidentaloma on various imaging. However, in the recent 2 decades, the latter proportion has increased to 30% and even 61% due to more frequent utilization of cross-sectional imaging techniques such as ultrasound, CT, and MRI (62, 63). Besides when incidentaloma is the starting point for the diagnosis, imaging usually follows as a next step after an established biochemical diagnosis of PPGL. The exception is biochemically negative PPGLs that occasionally can be detected by elevated chromogranin A. However, it has a low specificity due to interference of drugs, various diseases, and other tumors. Plasma or slightly less optimal 24-hour urinary metanephrines, measured by liquid chromatography with tandem mass spectrometry, form the cornerstone for the biochemical diagnosis (64). By measuring plasma-free metanephrines and 3-methoxytyramine (if available), a sensitivity and specificity of 98% and 94%, respectively, can be reached provided that proper preanalytic conditions for blood sampling are applied (65). Plasma metanephrines (normetanephrine or metanephrine), or methoxytyramine 2 or more times the upper reference limit or increases in 2 or more of all 3 metabolites suggest a high likelihood of a PPGL and patients are usually eligible to proceed to imaging studies (61). Adrenergic tumors are defined by an elevated plasma metanephrine of >0.31 nmol/L **and** by an increase in plasma metanephrine of >5% of the total increments in plasma normetanephrine and metanephrine beyond their upper reference limits (64). Tumors that do not satisfy these criteria are defined as noradrenergic or dopaminergic, the latter depending on the presence of a relative increase in methoxytyramine of >5% of the total increments in plasma normetanephrine, metanephrine, and methoxytyramine beyond their upper reference limits. In most patients, it will however be immediately clear from the actual plasma levels of metanephrines and methoxytyramine whether it is an adrenergic, noradrenergic, or dopaminergic tumor. This applies in particular when 1 metabolite is predominantly elevated and the others only slightly or not at all. In these latter patients, this calculation is redundant. However, in some patients with light to moderate elevations in 2 or 3 catecholamine metabolites, it may not be immediately clear what is the biochemical phenotype and in such patients this calculation might be helpful.

Results of plasma or urinary metanephrines are negative in the large majority of HNPGLs and may be false negative in patients with small PPGLs, and in rare cases of nonfunctional PPGLs (66). In these cases, the diagnosis may primarily rely on PPGL-specific imaging. This also applies when a patient presents with an acute cardiovascular emergency, possibly caused by PPGL, causing massive sympathetic activation and anyway (highly) elevated metanephrines. In certain emergency situations with patients admitted to the ICU or medium care, patients should be first stabilized and biochemical testing should be done at a later stage. However, if a diagnosis of PPGL is strongly suspected, imaging using contrast-enhanced CT or MRI in this context has the priority over biochemical testing. Under nonemergency circumstances, however, biochemical testing should precede imaging whenever a PPGL is suspected.

### Adrenal Incidentaloma

Although patients who are diagnosed with an adrenal incidentaloma have per its definition no symptoms or signs of catecholamine excess, that does not mean that the tumor is nonfunctional. Therefore, after a careful medical history and physical examination, in most patients, some symptoms and/or signs of catecholamine excess can be detected. Nevertheless, regardless of any clinical suspicion for PPGL, proper biochemical diagnosis to rule in or out the presence of PPGL, preferably by plasma metanephrines, remains mandatory (67). However, an exception should be made for incidentalomas with a low tissue density on unenhanced CT (Fig. 1). In general, PCCs display a density of ≥10 HU on unenhanced CT. Conversely, it is extremely rare that an adrenal incidentaloma with a low density of <10 HU is a PCC and virtually this low density excludes this tumor in nearly all patients with an adrenal incidentaloma (10, 11, 68). Three recent analyses have confirmed these data (69-71). One study showed a negative predictive value of nearly 100% for a PCC if the adrenal mass had a density of <10 HU. This suggests that in patients with an adrenal incidentaloma with a density of <10 HU, biochemical testing for PCC is not needed and work-up for this tumor is to be stopped here (69). An adrenal lesion that enhances stronger than 130 HU on CT after contrast is most likely a PCC and requires appropriate biochemical evaluation. On the other hand, contrast washout is often unreliable for the distinction between PCC and adrenal adenoma, since like in lipid-rich adenomas, a high (>60% absolute, >40% relative) washout is observed in one third of PCCs (Fig. 1) (10, 11, 72). In addition, there may be other features including calcification, cysts, necrosis, or hemorrhage that have been reported in patients with a PCC but these features per se lack sufficient diagnostic accuracy to play a decisive role in the differential diagnosis of an adrenal mass (73).

If an adrenal incidentaloma is detected on MRI, loss of adrenal signal intensity on opposed-phase gradient-echo images (chemical shift) has a high accuracy of 90% to 95% for lipid-rich adenoma and virtually excludes a PCC (74). Once an adrenal incidentaloma is detected on contrast-enhanced CT, additional MRI has no incremental diagnostic value in most patients.

### When a Biochemical Diagnosis of PPGL Is Established

In selected cases, anatomical imaging by CT or MRI may suffice to locate a PPGL and to proceed with surgery (75). When the epinephrine metabolite metanephrine is elevated either in plasma or urine, with or without an increase in normetanephrine, the tumor is likely to be located in the adrenal gland and anatomical imaging may initially be restricted to the upper abdomen. Additional body areas (chest, abdomen, pelvis, and head and neck) should be covered by anatomical imaging or complimented by the whole-body functional imaging in patients at higher risk of multifocal, extra-adrenal disease, metastatic disease, large tumor size (>5 cm), previous history of PPGL, or hereditary disorders other than MEN2 that is rarely associated with extra-adrenal and metastatic tumor presentation (76). With functional imaging, besides increasing sensitivity and properly locate a tumor, the added value often lies in improving specificity, indicating a high likelihood of PPGL.

Prospective studies on the actual added value of functional imaging in presurgical localization are lacking. The impact of ^123^I-MIBG SPECT on clinical decision-making has been evaluated in a large retrospective study (77). For the initial localization of PPGL, the addition of ^123^I-MIBG SPECT to CT/MRI improved the diagnostic accuracy only in rare cases at the cost of just as many incorrect interpretations in others (both false-positive and false-negative lesions), even when functional imaging was restricted to patients at risk for metastatic disease. Thus, the role of ^123^I-MIBG SPECT is now mainly limited to metastatic PPGLs for the purpose of treatment planning, for characterization of atypical and bilateral adrenal lesions and for distinguishing local recurrence from postoperative changes. Furthermore, semiquantitative ^123^I-MIBG SPECT may be useful to distinguish PCC from physiologic adrenal uptake (54, 78) or cortical adenoma (79).

The incremental value of functional imaging is probably higher for PET radiopharmaceuticals with a higher sensitivity for PPGL than for SPECT. These tracers provide high visual contrast between tumor and healthy tissue, which enables the detection of tumors that could potentially be missed by anatomical imaging. An exception may be a PGL in the urinary bladder wall, which can usually be well delineated by CT and MRI (80) but can be masked with functional imaging due intraluminal radiotracer accumulation that can be minimized if a patient empties the urinary bladder before imaging.

**Figure 6.**
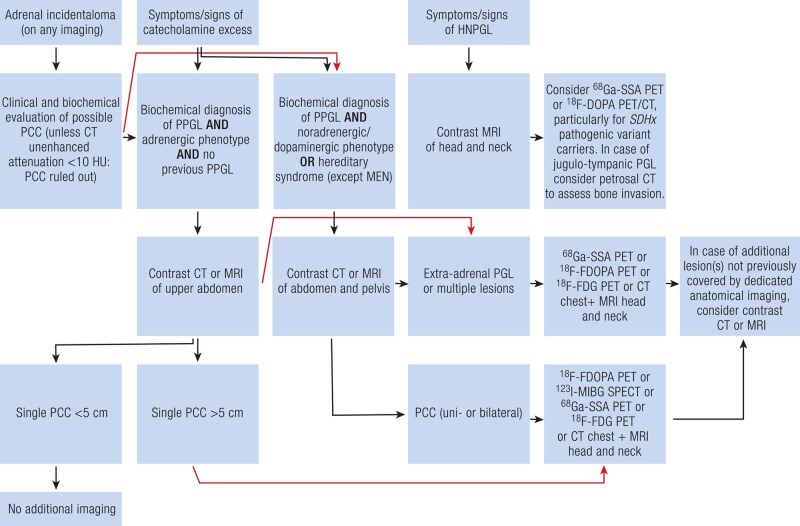
Imaging algorithm for initial diagnosis. Definitions: Plasma metanephrines (normetanephrine or metanephrine), or methoxytyramine 2 of more times the upper reference limit (61). Adrenergic tumors are defined by an elevated plasma metanephrine of >0.31 nmol/L **and** by an increase in plasma metanephrine of >5% of the total increments in plasma normetanephrine and metanephrine beyond their upper reference limits (63). Abbreviations: 3MT, 3-methoxytyramine; CT, computed tomography; FDG, fluorodeoxyglucose; FDOPA, fluorodihydroxyphenylalanine; HNPGL, head and neck paraganglioma; MEN2, multiple endocrine neoplasia type 2; MIBG, metaiodobenzylguanidine; MN, metanephrine; MRI, magnetic resonance imaging; NMN, normetanephrine; PCC, pheochromocytoma; PET, positron emission tomography; PPGL, pheochromocytoma/paraganglioma; SPECT, single photon emission computed tomography; SSA, somatostatin analogue.

**Figure 7.**
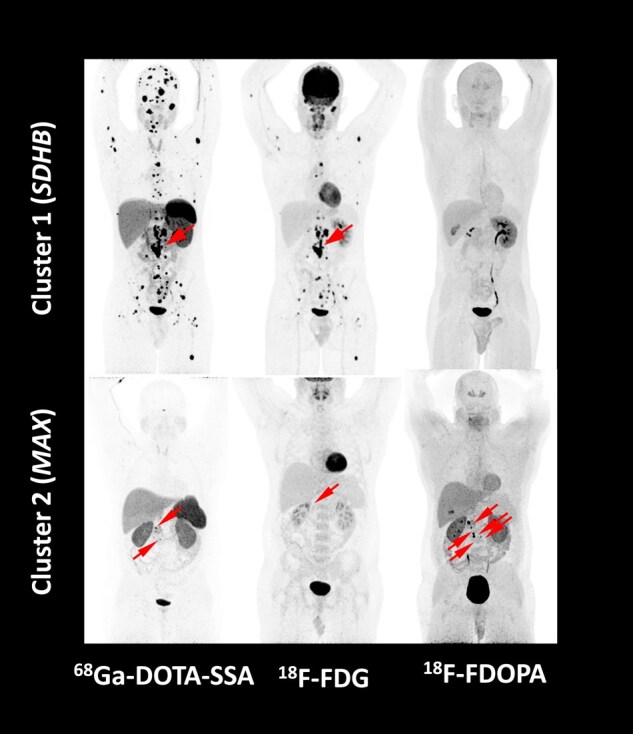
Impact of genetic cluster on functional imaging. PET images of a 20-year-old male with cluster 1 PPGL related to an *SDHB* pathogenic variant (upper panels) and of a 38-year-old male with PCC related to a cluster 2 *MAX* pathogenic variant. Abbreviations: FDG, fluorodeoxyglucose; FDOPA, fluorodihydroxyphenylalanine; *MAX*, MYC-associated factor X; *SDHB*, succinate dehydrogenase B; SSA, somatostatin analogue.

The choice of a radiopharmaceutical depends heavily on tumor genotype and biology (81, 82) as well as location (adrenal vs extra-adrenal), size, and biochemical phenotype. A tailored functional imaging approach is detailed in the updated European Association of Nuclear Medicine Practice Guideline/Society of Nuclear Medicine and Molecular Imaging Procedure Standard 2019 (83). In general, ^68^Ga-DOTA-SSA PET/CT is the most sensitive approach for PPGL localization and can serve as a starting point for functional imaging in most cases (84), including children (85, 86). In a systematic review and meta-analysis, the pooled detection rate of PPGL ^68^Ga-DOTA-SSA PET/CT was 93% (95% CI 91-95%), which was significantly higher than that of ^18^F-FDOPA PET/CT (80%, 95% CI 69-88%), ^18^F-FDG PET/CT (74%, 95% CI 46-91%), and ^123/131^I-MIBG scintigraphy (38%, 95% CI 20-59%, *P* < .001 for all) (87). This meta-analysis is hampered by mixing PCCs of various origin and genotypes. Head to head comparison between ^68^Ga-DOTA-SSA PET/CT and ^18^F-FDOPA PET/CT has been performed in only 7 studies. Overall, ^68^Ga-DOTA-SSA is the most sensitive tracer for HNPGLs, as well as for *SDHx* PPGLs (Figs. 6 and 7). This is important since in clinical practice, tumor staging often precedes the results of genetic testing. Partly due to physiological adrenal uptake of ^68^Ga-DOTA-SSA PET/CT, more tumor specific ^18^F-FDOPA PET/CT may be preferred in PCCs that are small and/or related to known mutations in *NF1*, *RET*, *VHL*, or *MAX* (55, 81, 88). If neither of these modalities is available, alternatives are either ^123^I-MIBG SPECT or ^18^F-FDG PET/CT, the latter particularly for extra-adrenal and/or *SDHx* PPGLs (83). In patients presenting with polycythemia/PPGL syndromes due to mutations in *EPAS1* or prolyl hydroxylases, ^18^F-FDOPA PET/CT is superior to ^18^F-FDG PET/CT and ^68^Ga-DOTA-SSA PET/CT (89).

In contrast, when the genotype of an established PPGL is unknown, or germline variants are of unknown pathogenicity, functional imaging can assist in tumor characterization. An example is the distinct ^18^F-FDG accumulation in *SDHx* PPGLs as compared to other genotypes due to accelerated glucose phosphorylation by hexokinases (90-93). An alternative experimental approach is that of proton MR spectroscopy for in vivo succinate detection as a specific and sensitive hallmark of SDH deficiency in PPGL (27, 94, 95).

Compared with adults, PPGLs in children are more frequently hereditary (80%), extra-adrenal (66%), multifocal (32%), and at high risk for metastatic disease (49%) (96). Therefore, whole body functional imaging seems well justified. ^68^Ga-DOTA-SSA PET/CT is now suggested as the initial functional imaging modality of choice in pediatric cases (97). However, when frequent follow-up scanning is anticipated, for instance in the context of hereditary cases or metastatic disease, MRI with contrast could be used instead to reduce cumulative radiation burden (98), bearing in mind, however, its limited sensitivity for pulmonary metastases.

### Head and Neck Paraganglioma

Anatomical imaging of HNPGL is preferably performed by MRI. MRI can be complimented by CT to assess bone invasion, which is especially useful for jugulo-tympanic PGLs. Functional imaging helps to distinguish HNPGLs from other tumor entities, to detect small additional (metastatic) lesions in the head and neck, and to screen for additional thoracic/abdominal PGLs. Both ^18^F-DOPA PET/CT and ^68^Ga-DOTA-SSA PET/CT are suitable for this purpose; the latter being preferred in the setting of *SDHx* HNPGL.

## Clinical Scenario II: Postsurgical Follow-up and Tumor Surveillance in Asymptomatic Mutation Carriers

### Postsurgical Follow-up

Tumor surveillance after any surgical intervention is crucial as a literature review showed that the prevalence of recurrence (new tumor, local or metastatic) within 5 years after surgery was around 5% with a rate of 1% every year after intervention (99). A higher prevalence was found in an evaluation of a large European database (European Network for the Study of Adrenal Tumors (ENSAT)) with an overall risk of a new event within 5 years of 8% to 14% after successful primary surgery (100). In a recent study, in 29.1% and 17.7% of patients with sporadic PPGL, recurrences were respectively diagnosed at least 10 and 15 years after first diagnosis (101). The risk of recurrence is higher for sympathetic PGL than PCC, higher in patients with a known pathogenic variant of one of the PPGL-related susceptibility genes than in sporadic cases and higher in patients with large initial tumor size (99, 101).

**Figure 8.**
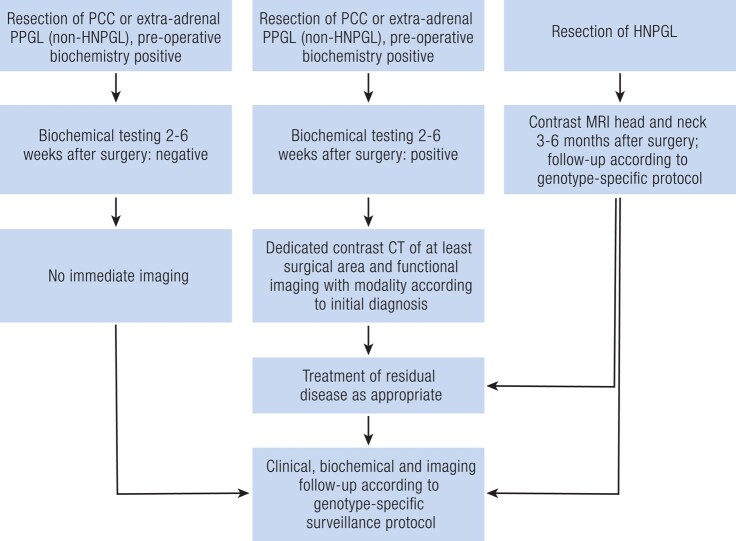
Imaging algorithm for postsurgical follow-up. Abbreviations: CT, computed tomography; HNPGL, head and neck paraganglioma; MRI, magnetic resonance imaging; PCC, pheochromocytoma.

In general, if biochemical test results normalize within the first 6 to 8 weeks after surgery, no additional imaging is required as a residual tumor mass is considered as excluded (Fig. 8). However, in patients with preoperative biochemical-negative tumors without metastases, imaging is indicated at 3 to 6 months after surgery to check for residual tumor mass. In patients who have undergone surgery for an apparently sporadic or a hereditary PPGL that is not related to *SDHx* pathogenic variants and who had preoperatively positive biochemical test results, annual long-term follow-up should be performed by biochemical testing, with subsequent cross-sectional imaging only in those cases with positive biochemical test results (100). For assessment of local recurrence, MRI should be avoided in case of presence of surgical clips that inevitably cause artifacts.

For patients who have undergone surgery for an *SDHx* PPGL, lifelong periodic imaging to support detection of extra-adrenal sympathetic PGLs or HNPGLs is indicated every 2 to 3 years, irrespective of biochemical test results. In patients in whom metastatic PPGL is diagnosed at primary surgery, mode and intensity of follow-up is dictated by signs and symptoms, tumor burden and rate of disease progression. This may include cross-sectional whole-body imaging by CT or MRI and/or even functional imaging (61). A similar approach can be used for patients with somatic *HIF2A* pathogenic variants since they have a very high rate of multifocality, recurrence, and metastasis (102).

### Tumor Surveillance in Asymptomatic Mutation Carriers

With the availability and decreasing costs of testing for germline and somatic pathogenic variants, life-long surveillance using biochemical testing and imaging can be individually tailored based on the underlying pathogenic variant of PPGL susceptibility gene (83, 100, 103). This applies to asymptomatic relatives of index patients (patients who had a previous PPGL) who carry a germline pathogenic variant of a gene of cluster 1 PPGLs (here particularly *SDHx*). These subjects are candidate for long-term clinical, biochemical, and imaging surveillance because of their high potential for local and metastatic recurrences (12, 101, 103). It is crucial that these subjects are identified before the PPGL becomes symptomatic. For some genes of the Cluster 1 PPGLs such as *VHL* and *SDHx*, it has been shown that early identification of pathogenic variants of these genes has a relevant positive impact on management of these patients (104). More importantly, recent retrospectively collected data suggest that subsequent periodic surveillance of asymptomatic carriers of *SDHB* pathogenic variants results in early tumor detection with improved clinical outcome (105). It should also be noted, that those data published about surveillance included heterogeneous biochemical and imaging test procedures. The latter included merely MRI imaging, ^68^Ga-DOTATATE PET/CT or ^18^F-FDG-PET/CT at rather varying time intervals.

As initial screening of asymptomatic carriers of *SDHx* pathogenic variants, measurement of blood pressure and plasma or urinary metanephrines together with the whole-body MRI has been recommended for children, with ultrasound as an alternative only if MRI is not feasible or available (12). A similar screening repertoire has been recommended for adult carriers of pathogenic variants except for addition of PET/CT. However, this latter addition is not supported by strong agreement among experts. Furthermore, under certain circumstances (insurance approval, cost) or based on a patient's request, CT is performed instead of MRI.

Furthermore, follow-up surveillance after negative initial screening needs to be performed lifelong for *SDHx* pathogenic variants. In addition to annual clinical examination (blood pressure), measurement of plasma or urinary metanephrines can be carried out every 2 years in children whereas the latter is recommended annually in adults (12). In addition, total body MRI is advised be repeated every 2 to 3 years in both children and adult carriers. To limit radiation exposure in patients vulnerable for radiation-induced gene mutations, total body MRI is the preferred imaging tool for long-term follow-up in asymptomatic carriers of *SDHx* pathogenic variants, both in children and adults. In addition, because of the risk of gadolinium accumulation in certain brain areas at repeated administration (106), it could be considered to use no contrast agent in follow-up MRIs after the initial MRI with gadolinium is used, although this likely compromises sensitivity.

For carriers of *SDHx* pathogenic variants functional imaging is only indicated in adults, although consensus among experts is weak. If functional imaging is considered, ^68^Ga-DOTA-SSA PET/CT has been recommended as first choice in this group of patients (83).

Although periodic surveillance is also important for Cluster 2 PPGLs, including those with pathogenic variants in *RET*, *NF1*, *MAX*, *TMEM127*, *HRAS*, and *FGFR1*, there is likely no role for imaging as surveillance on top of negative biochemical testing. This probably also pertains to patients with Cluster 1 gene mutations *HIF2A*, *VHL*, and *FH*, although periodic imaging may be anyway indicated to screen for non-PPGL tumors.

## Clinical Scenario III: Metastatic PPGL: Staging, Follow-up and Theranostics

### Staging of Metastatic PPGL

A minority of patients with PPGL, between 10% and 17%, develop metastatic disease. In around half of these patients, metastases are already present at the initial diagnosis (“synchronous”), whereas metachronous metastases may develop in the course of months, years or decades after surgery. Predictors of metastatic development include large tumor size, extra-adrenal location, noradrenergic/dopaminergic phenotype, and presence of *SDHB* pathogenic variants. The TNM staging for PPGL has adopted a cut-off of 5 cm as well as extra-adrenal location as important determinants of the risk of metastasis (107). It is impossible to distinguish “malignant/metastatic” from “benign” PPGL based on histopathologic or molecular features. Capsular and lymphovascular invasion, coarse nodularity, the presence of atypical nuclei, and a high proliferation index (Ki67) are predictive of metastatic behavior but are unreliable to determine prognosis in the individual patient. According to the 2017 World Health Organization classification of endocrine tumors, all PPGLs have to be considered to have metastatic potential. Metastatic PPGL is formally defined by the presence of metastatic lesions in tissues where chromaffin cells are normally absent (ie, lymph nodes and bones) (108). Besides in lymph nodes (80%) and the skeleton (72%), distant metastases mainly occur the liver (50%), and lungs (50%) (109). Metastases to the brain, breasts, skin, and ovaries have been described in rare cases.

**Figure 9.**
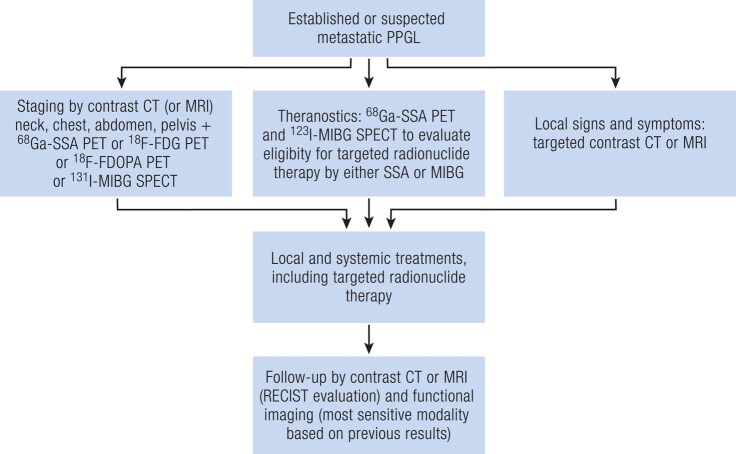
Imaging algorithm for metastatic disease. Abbreviations: CT, computed tomography; FDG, fluorodeoxyglucose; FDOPA, fluorodihydroxyphenylalanine; MIBG, metaiodobenzylguanidine; MRI, magnetic resonance imaging; PET, positron emission tomography; PPGL, pheochromocytoma/paraganglioma; SPECT, single photon emission computed tomography; SSA, somatostatin analogue.

For the purpose of initial staging and comprehensive localization of metastases, combining anatomical with functional imaging is regarded as essential. ^68^Ga-DOTA-SSA PET/CT is designated as the first-choice imaging modality, regardless of the genetic background (Fig. 9) (53, 83, 110). ^18^F-FDOPA PET/CT provides an excellent alternative, although its sensitivity is slightly less in the presence of *SDHx* mutations (39, 53). Specifically in case of common *SDHB* metastatic PPGL, ^18^F-FDG PET/CT is preferred over ^18^F-FDOPA PET/CT when ^68^Ga-DOTA-SSA PET/CT is not available (90, 111). When using ^18^F-FDG, avid uptake by brown adipose tissue should be not mistaken for metastases (112-114). As third choice, ^123^I-MIBG SPECT/CT can be applied, but this tends to underestimate the extend of metastatic disease due its clearly lower sensitivity as compared to PET in most cases (115). ^123^I-MIBG SPECT/CT does have an important role in the context of theranostics (see below).

When using CT of chest, abdomen, and pelvis for tumor staging, bone lesions may remain undetected, either because they are located outside the field of view or because of their small size and lack of an adjacent soft-tissue component. In general, bone lesions are better detected by ^68^Ga-DOTA-SSA PET/CT and ^18^F-FDG PET/CT (90, 110). In case of a spinal PPGL lesion on PET/CT, MRI of the spine should be performed to assess vertebral stability, to anatomically delineate the tumor as well as to rule out impending myelum compression (42). The first-choice imaging modality for detection of liver metastases is MRI with gadolinium.

### Follow-up and Response Monitoring of Metastatic Disease

Personalized treatment of metastatic PPGL should mirror its specific molecular signature, as recently reviewed by Nölting et al (103). Optimal management requires a multidisciplinary specialized team. Goals are to reduce tumor size, catecholamine-related adverse events, and prevent tumor progression. In case of limited metastatic disease such as local metastatic lymphadenopathy, surgical metastasectomy can be undertaken. Other local therapies such as external beam irradiation, radiofrequency ablation, tumor embolization, cryotherapy, and percutaneous microwave coagulation can be applied for local symptom relief. Regarding systemic treatment, patients likely to benefit from chemotherapy are those with rapidly progressive disease. The best examined protocol is a combination of cyclophosphamide, vincristine, and dacarbazine (116). As an oral alternative, temozolomide has been successfully applied to treat *SDHB* PPGL (117). For less progressive disease, radionuclide therapy can be applied as detailed below. In subsets of patients, antiangiogenic treatment with tyrosine kinase inhibitors such as sunitinib has been associated with partial response, disease stabilization, and improved blood pressure control (118). Other targeted treatments are under investigation.

Traditionally, tumor response monitoring is performed by measuring changes in tumor size on anatomical imaging according to Response Evaluation Criteria in Solid Tumors (RECIST) 1.1 (119). Response to systemic treatment in metastatic PPGL is usually achieved by CT according to RECIST. However, metabolic changes often precede any measurable change in tumor size, making early response assessment difficult with such morphological measurements. Additionally, targeted therapies may induce necrotic or cystic changes that do not result in tumor tissue shrinkage. Furthermore, bone metastases without soft tissue measurable component are not considered RECIST assessable, further complicating PPGL response assessment using RECIST (120). This suggests that PET-based response evaluation, either with ^68^Ga-DOTA-SSA PET/CT, ^18^F-FDOPA PET/CT, or ^18^F-FDG PET/CT, might be more accurate, given their higher sensitivity and the fact that bone lesions are included. In several other malignancies it has been demonstrated that assessment of metabolic response by ^18^F-FDG PET/CT is more accurate for tumor response assessment than RECIST and correlates better with patient outcome (121). Currently, 2 sets of criteria to quantify metabolic response are available: PET Response Criteria in Solid Tumors (PERCIST) 1.0 (122) and the criteria developed by the European Organization for Research and Treatment of Cancer (EORTC) (123). Both sets are being validated in prospective clinical studies, including PPGL.

### Theranostics and Targeted Radionuclide Therapy

#### Principles

Theranostics is the combination of 2 terms: therapeutics and diagnostics. This can be achieved with a single radiopharmaceutical that emits both therapeutic and diagnostic particles. The prime example of this approach is radioiodine theranostics where the results from post-therapy scan together with pathological and molecular genetic findings can determine whether an individual patient is likely to benefit from additional cycles of radioiodine treatment. The term theranostics has evolved and now is used for describing the combination of using 1 radiopharmaceutical to select patients for targeted radionuclide therapy (TRT), followed in positive cases by the use of a second radiopharmaceutical that contain the same targeting molecule labeled with a therapeutic isotope such as ^177^Lu to treat the main tumor and any metastatic lesions. Both radiopharmaceuticals are called a theranostic pair. TRT relies on tumor-specific characterization tightly linked to appropriate therapy selection and individualized medicine. Since PPGL can overexpress SSTR and/or NET, they can potentially be treated by radiolabeled somatostatin analogs or ^131^I-MIBG. In practice, both radiopharmaceuticals can be considered based on the radionuclide uptake for each tracer, favoring the one that is clearly superior in targeting most or all the patient's lesions.

TRT is a radiotherapeutic option that induces DNA damage and apoptosis. From a radiobiological standpoint, PPGLs share some characteristics with radioresistant tumors with a well-differentiated nature and a slow turnover rate and probably robust DNA repair mechanisms. Additionally, there are some features that may limit the tumoricidal effect of TRT such as large tumor volume and necrotic/hemorrhagic/cystic degeneration that may reduce radiopharmaceutical accessibility and induce heterogeneous dose deposit. Together these data explain that most PPGLs exhibit disease stabilization following TRT. The potential impact of pseudo-hypoxia associated with an *SDHx* genotype is unknown. As a general concept for TRT, several factors can influence treatment response such as heterogeneity of tumor target expression, organ distribution of the disease, absorbed dose and tumor mutational factors.

The targeted population that is suitable for TRT include patients with PPGL with metastatic/inoperable/progressive disease and/or those with uncontrolled catecholamine excess-related signs and symptoms (such as hypertension, arrhythmias and other cardiovascular events).

#### ^131^I-MIBG therapy

In the past, conventional or low specific activity (LSA) ^131^I-MIBG (0.555-1.850 GBq/mg) was used (124). More recently, a high specific activity (HSA) ^131^I-MIBG (92.5 GBq/mg) has been approved by the FDA and is the standard commercially available product in the United States. Since HSA contains 99% of radioactive MIBG, there is no competition with nonradioactive MIBG for the NET-related transporter. Compared with LSA, the potential clinical benefit of using HSA is expected for tumors that strongly express NET (target) and have an efficient storage system, otherwise the antitumor effect remains very limited. Recent phase I and II trials of high specific activity HSA ^131^I-MIBG (iobenguane I-131, Azedra) have been conducted in patients with PPGLs (125, 126). In the phase II study, 68 patients were treated with approximately 18.5 GBq/cycle over 2 cycles. The primary endpoint was reached with reduction in baseline antihypertensive medication lasting ≥6 months in 25% of patients. Using at least 1 single treatment, partial response and stable disease rates within 12 months of 23% and 69%, respectively were documented for a total disease control rate of 92%. Median overall survival (OS) was 37 months. No patients had acute hypertensive events, but 72% (49/68) of patients experienced grade ≥3 hematotoxicity (41% thrombocytopenia, 41% leukopenia, 38% neutropenia, and 21% anemia) and 25% (17/68) required hematologic supportive care. Myelodysplastic syndrome was observed in 4% and secondary malignancies in 3% (acute myeloid leukemia and acute lymphocytic leukemia in 1 patient each). In practice, *Azedra* regimen requires a dosimetric step at each cycle.

#### Peptide receptor radionuclide therapy

Peptide receptor radionuclide therapy (PRRT) with radiolabeled somatostatin analogs has been rapidly implemented into the therapeutic arsenal for PPGL. A meta-analysis of PRRT in metastatic PPGLs, regardless of pathogenic gene variants concluded that there was a beneficial effect using either ^90^Y or ^177^Lu-DOTATATE, with an objective response rate of 25% (127). While some studies have utilized ^90^Y-DOTATATE, it is not FDA approved nor is it readily available and has greater risk of renal toxicity; as a result, most studies in the world will continue to use ^177^Lu-DOTATATE. Of note, ^177^Lu-DOTATATE is also not yet FDA approved for PPGL. Adverse effects include acute/subacute hematological cytopenia (4-11% grade 3-4, mostly reversible) and delayed complications such acute myeloid leukemia. and myelodysplastic syndromes (2-4%). They mainly occur after a median period of 26 months and prolonged thrombocytopenia (>3 months) should raise the suspicion of therapy-related myeloid neoplasms. The latter have a poor prognosis and allogeneic stem cell transplantation remains the only potentially curative regimen. Nephrotoxicity is a rare event.

As previously stated, the therapeutic responses obtained to PRRT are mainly disease stabilization (in previously progressive disease) (81, 127-131). This is mainly related to large tumor load that needs to be targeted by PRRT, and possibly the low-dose regimen per gram of tumor tissue. A major feature of PRRT is the possibility following in vivo the fate of a radioactive tracer and to evaluate the absorbed doses to the tumor and organs at risk. Regarding tumor dosimetry, this is less easy to calculate, compared with external radiotherapy since the cells and organs are irradiated not only for seconds or minutes, but continuously over a long period of time with permanently changing dose rate. Furthermore, the relationships between absorbed dose and response rate are not well-established. In order to increase the absorbed dose while preserving organs at risk, 2 main options that have been proposed: the standard 7.4 GBq/cycle with variation of number of cycles until preset biological effective doses to the kidney and bone marrow are reached or a fixed 4 cycles regimen with variable activity per cycle to reach the same dose limits. The development of clinical dosimetry is made of several steps that need to be optimized to provide validated results. Data obtained from the clinical study will enable the evaluation of absorbed doses to tumors and tissues/organs and compared with clinical outcomes. Few clinical-ready workflows have been developed for lutetium-based therapies, from image analysis to dosimetry computation and treatment report.

#### Missing data

Data of comparative outcomes of LSA and HSA ^131^I-MIBG vs PRRT in PPGL are limited. It is not clear that 1 reagent is clearly superior when patients are selected based on uptake specificity. For MIBG, there is no study that directly compare the efficacy or outcomes of treatment with the approved HSA regimen compared with the LSA treatment with repeated cycles. There are no data that support any difference in terms of response to TRT across genotypes.

## Future Perspective

### Diagnostics

Beyond glucose, there is an expanding metabolic repertoire that is needed to fuel cancer cells and increase biomass. Glutamine metabolism that is found to be abnormal in *SDHx* PPGLs can be indirectly studied by glutamine imaging or by ^18^F-fluciclovine and may guide the potential use of innovative treatments such as glutaminase inhibitors (132-134). New theranostic pairs may also emerge as new opportunities for TRT such as prostate-specific membrane antigen, which is expressed in the tumor vasculature of some PPGLs (135). New PET tracers that target NETs ^124^I-MIBG can detect PCC with a high accuracy and can be used as a companion agent for selecting candidates for ^131^I-MIBG (136). ^18^F-meta-Fluorobenzylguanidine is also a promising alternative PET tracer to ^123^I-MIBG (137). Beyond new tracers, nuclear medicine can also provide imaging biomarkers. Radiomics allows to extract information from images beyond the capabilities of the human eye. This is a multistep process that uses machine (deep) learning and mathematical predictive models. In the setting of PPGL, radiomics has been evaluated for disease subtyping (cluster 1 vs others) (138, 139) and has shown promising results. There are still however some challenges mainly due to lack of standards, limited reproducibility and the lack of fully automated segmentation methods. Radiomics signatures still require to be validated in the setting of multicentric studies. All of the validated multiomics data from various modalities together with critical clinical information on the disease and treatment responses could be implemented into secured databases and help to treat some patients that share some similarities (also called digital twins). Many international initiatives led by governments or companies with consortium partners have been initiated to support such projects in oncology and are of particular interest for rare diseases.

### Theranostics and TRT

In PPGL, the use of SSTR antagonists that display higher occupancy and more prolonged binding to SSTR may also increase >10 times higher uptake than agonists and may represent an interesting alternative approach to agonists (140, 141). Manipulation of target expression is also an attractive approach such as use of histone deacetylase inhibitors (142).

Beyond the target, alpha emitters that have 100 to 1000 times greater linear transfer energy than beta emitters and therefore more cytotoxic, have emerged as an attractive alternative treatment option to beta emitters in PRRT. In a recent study including 9 patients with PPGL using actinium-225–labeled SSA, partial response was observed in 50% of cases, with an overall response in 87.5% (143). Seven out of 9 patients had received previous ^177^Lu-based PRRT. A reduction or interruption of antihypertensive drugs was achieved in 3/7 and 2/7, respectively. Interestingly, no high-grade toxicity occurred. More recently, data from the phase I using Pb212-labeled SSA has shown an overall response of 80% in neuroendocrine tumors (no PPGL) and no serious treatment-emergent adverse events (144). Of note, Pb212 acts as in vivo generator of α-particles. These reassuring results in term of side effects will probably give a greater impetus toward the use of targeted α-particle therapy in patients with PPGL.

Based on preclinical studies, other radionuclides, notably terbium-161, that combine the β- and Auger emissions also appear promising, especially so when coupled to SSTR antagonist, probably leading to substantial damage to cell membranes of tumor cells and activation of signal transduction pathway (ceramide-mediated) leading to apoptosis (145).

There is also a lot of exciting work on the combination therapies such as radiosensitizers or immunotherapy. The role of TRT would be to allow local control, initiate T cell priming, and turn out “cold to hot” tumor that together with immune checkpoint blockade could improve outcomes of cancer patients. In this context, immune-PET can help to monitor real-time T cell infiltration that can be useful for synergizing treatments.

Since therapy-related myeloid neoplasms represent the most dreaded complications of TRT, it is hoped that in near future that the improved genomic characterization of a patient's genetic background will allow to determine an individualized risk for such complications and guide proper treatment decisions.

## Abbreviations

CT: computed tomography
DOTA: dodecane tetraacetic acid
FDG: fluorodeoxyglucose
FDOPA: fluorodihydroxyphenylalanine
HSA: high-specific activity
LAT: L-amino acid transporter 1
LSA: low specific activity
MIBG: metaiodobenzylguanidine
MRI: magnetic resonance imaging
NET: norepinephrine transporter
PCC: pheochromocytoma
PET: positron emission tomography
PPGL: pheochromocytoma/paraganglioma
PRRT: peptide receptor radionuclide therapy
SDH: succinate dehydrogenase
SPECT: single photon emission computed tomography
SSA: somatostatin analogue
SSTR: somatostatin receptor
TRT: targeted radionuclide therapy
