# Correction to: “Imaging of Pheochromocytomas and Paragangliomas”

**DOI:** 10.1210/endrev/bnaf023

**Published:** 2025-08-08

In the above-named article by Timmers HJLM, Taïeb D, Pacak K, Lenders JWM (*Endocrine Reviews* 2024; 45(3): 414–434; doi: 10.1210/endrev/bnae001), there was an error in the Disclosures section.

In the above-mentioned article, in the Disclosures section, the disclosures originally read, “The authors declare that there is no conflict of interest that could be perceived as prejudicing the impartiality of the research reported.” In the corrected article, the disclosures should read, “D.T. is a cofounder, stakeholder, and the chief medical officer (CMO) of SILON Therapeutics. All other authors have nothing to declare.”

doi: 10.1210/endrev/bnae001

